# A Rare Case of Brain Abscesses Caused by Acremonium Species

**DOI:** 10.7759/cureus.14396

**Published:** 2021-04-10

**Authors:** Armeena Anis, Fnu Sameeullah, Jamil M Bhatti

**Affiliations:** 1 Medicine, Dr. Ziauddin Hospital, Karachi, PAK; 2 Internal Medicine, Carney Hospital, Boston, USA; 3 Infectious Diseases, The Cancer Foundation Hospital, Karachi, PAK; 4 Infectious Diseases, Dr. Ziauddin Hospital, Karachi, PAK

**Keywords:** acremonium, voriconazole, amphotericin, recurrence, brain abscess, fungal abscess

## Abstract

*Acremonium* species are saprophytic fungi that are rarely pathogenic in humans. According to several reports, *Acremonium* species can cause various diseases, ranging from superficial infections after traumatic inoculation in immunocompetent individuals to invasive infections in the immunocompromised. To the best of our knowledge, this is the first case report of brain abscess in an 18-year-old male caused by *Acremonium* species in Pakistan. A combination of intravenous amphotericin B and oral voriconazole was administered to the patient, which resulted in marked clinical improvement. However, the recurrence of fungiwas observed after three months of completion of the antifungal course. The purpose of this report is to alert clinicians regarding this pathogen and its ability to cause systemic disease.

## Introduction

The genus *Acremonium*, previously known as Cephalosporium, consists of approximately 150 species of hyaline filamentous fungi. Most of these species are environmental saprophytes that are primarily isolated from soil and decaying plant material [1]. While fungi belonging to the genus *Acremonium* rarely infect humans; however, when they do, they can infect both immunocompetent and immunocompromised individuals [2]. Several cases of localized and systemic infections involving multiple organs, including the brain, have been reported to be caused by these fungi [3]. Here, we report an unusual case of brain abscesses caused by the *Acremonium* species in Pakistan.

## Case presentation

An otherwise healthy 18-year-old male experienced a near-drowning incident in fresh water in June 2020. He was taken to a local hospital after being rescued and resuscitated. He was then treated in the intensive care unit for aspiration pneumonia and was put on ventilator support. Four days later, as the patient’s condition improved, he was extubated with a Glasgow Coma Scale score of 15 and oxygen (O_2_) saturation of 99% on room air. The patient was discharged after two more days. After a week, he presented to Dr. Ziauddin Hospital with a temperature of 101°F and a one-day history of shortness of breath at rest. During his physical examination, he appeared to be conscious and restless and was found to have tachypnea, temperature of 102°F, pulse of 108 beats/min, respiratory rate of 26 breaths/min, a blood pressure (BP) of 115/75 mmHg, and O_2_ saturation of 90% on room air. His chest examination revealed harsh vesicular breathing. The rest of the clinical examination was unremarkable.

On admission, laboratory investigations revealed a high total leukocyte count of 20.0 × 10^9^/l, procalcitonin level of 19.6 ng/ml, erythrocyte sedimentation rate of >100 mm/h, and D-dimer level of 3,144 ng/ml fibrinogen equivalent units (FEU), in addition to a markedly elevated C-reactive protein level of 440.05 mg/l (Table 1).

**Table 1:** Baseline laboratory values on the day of admission Hb, hemoglobin; HCT, hematocrit; TLC, total leukocyte count; ESR, erythrocyte sedimentation rate; AST, aspartate aminotransferase; ALT, alanine aminotransferase; GGT, gamma-glutamyl transferase

Variables	Normal Range	Value on Admission
Hb (g/dl)	13.0–17.0	12.3
HCT (%)	40–50	36
TLC (× 10^9^/l)	11.5–14.5	20.0
Platelets (× 10^9^/l)	150–440	798
ESR (mm/h)	0–20	>100
Sodium (mEq/l)	136–145	137
Potassium (mEq/l)	3.5–5.1	2.9
Chloride (mEq/l)	98–107	102
Bicarbonate (mEq/l)	23–29	23
Urea (mg/dl)	17–49	25
Creatinine (mg/dl)	0.9–1.3	0.74
C-reactive protein (mg/l)	<5.0	440.05
Procalcitonin (ng/ml)	<0.046	19.6
D-dimer (ng/ml FEU)	55–1,550	3,144
Albumin (g/dl)	3.50–5.20	3.12
Total bilirubin (mg/dl)	0–2	0.45
Direct bilirubin (mg/dl)	<0.2	0.21
AST (U/l)	<35	23
ALT (U/l)	<45	27
GGT (IU/l)	<55	30
Alkaline phosphatase (U/l)	53–128	55

The chest X-ray revealed bilateral patchy infiltrates. Although the patient’s blood culture was sterile, his sputum culture revealed two micro-organisms, namely *Enterobacter* species and *Pseudomonas aeruginosa* (Table 2).

**Table 2:** Sputum culture and sensitivity report revealing the presence of Enterobacter species (micro-organism 1) and Pseudomonas aeruginosa (micro-organism 2) R, resistant; S, sensitive

Antibiotics	Micro-organism 1	Micro-organism 2
Amikacin	R	
Aztreonam	R	S
Cefoperazone/sulbactam	R	
Cefixime	R	
Ceftazidime	R	S
Ceftriaxone	R	
Colistin	S	S
Cotrimoxazole	R	
Gentamicin	R	S
Imipenem		S
Meropenem	R	S
Ofloxacin	R	S
Ciprofloxacin	R	S
Polymyxins	S	S
Tazobactam/piperacillin	R	S

**Figure 1:**
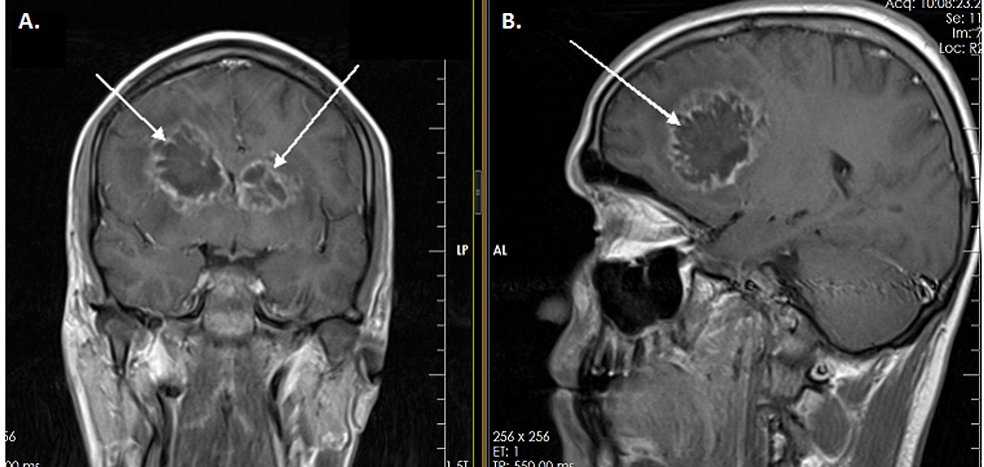
Magnetic resonance images of the brain (without contrast) showing arrows pointing to multifocal, lobulated, ring-enhancing lesions in both cerebral hemispheres surrounded with vasogenic edema (A, B)

**Figure 2:**
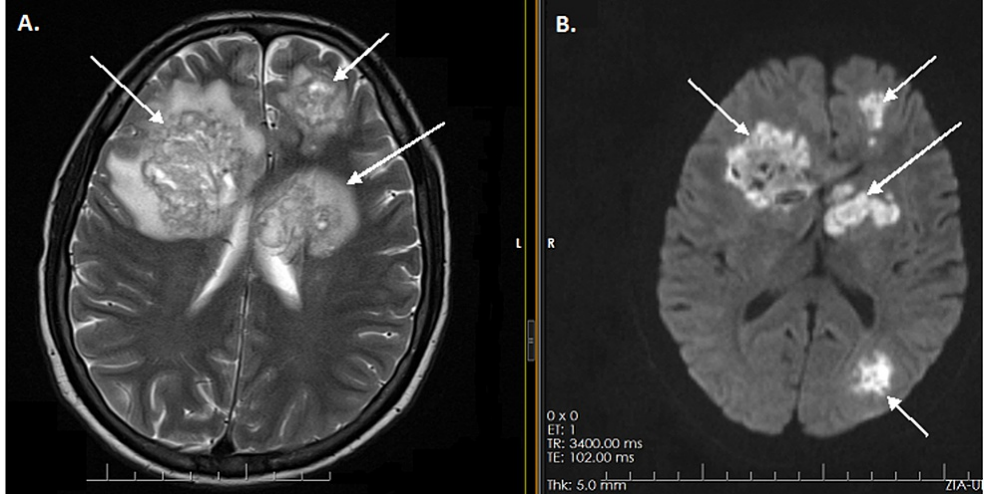
Diffusion-weighted magnetic resonance images of the brain with arrows pointing out multiple lesions with restricted diffusion in both cerebral hemispheres, including the basal ganglia, left thalamus, left cerebral peduncle, and right frontal lobe, surrounded with vasogenic edema (A, B)

Treatment with inhaled and intravenous colistin was initiated. However, despite the micro-organisms being sensitive to colistin, no clinical improvement was noted, and the patient remained febrile. Blood culture repeated on the 12th day of admission revealed the presence of *Acinetobacter* species sensitive to colistin. Hence, the administration of colistin was continued. On the 15th day of admission, the patient became drowsy and lethargic and developed persistent pyrexia of 104°F. Therefore, magnetic resonance imaging (MRI) of the head was performed and revealed multiple brain abscesses. The largest one was present in the right frontal lobe and was associated with vasogenic edema (Figures 1A, 1B, 2A, 2B).

**Figure 3:**
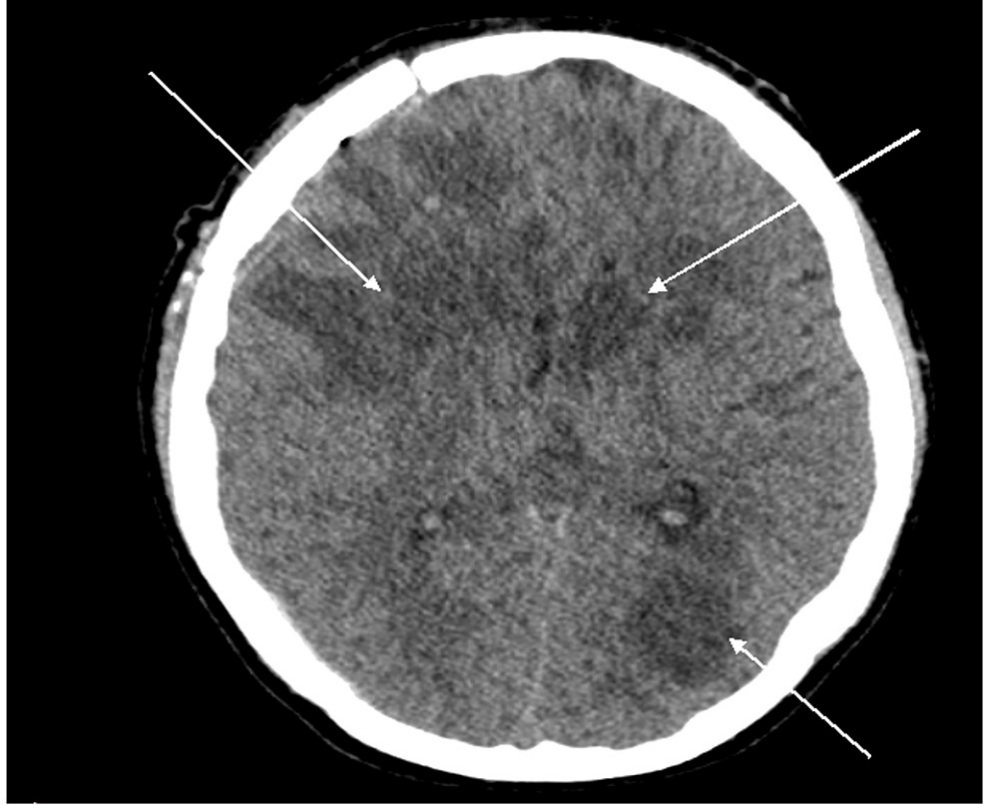
Post-craniotomy computed tomography scan of the brain showing partial evacuation of the right frontal lobe lesion and multifocal hypodense lesions as shown by arrows in both cerebral hemispheres

Consequently, craniotomy and drainage of purulent material from the right frontal abscess were performed. Moderate growth of *Acremonium* species was noted in the pus extracted during the surgery. Intravenous antifungal therapy with amphotericin B (30 mg once a day) was initiated. However, the patient still remained febrile and drowsy. The antifungal regimen was therefore modified, and voriconazole injection (6 mg/kg twice a day for the first 24 h and 4 mg/kg twice a day for the next two days) was added. This was later switched to oral voriconazole (200 mg twice a day). After administering voriconazole, the patient became afebrile and his neurological status improved. A post-craniotomy computed tomography (CT) scan of the brain revealed partial evacuation of the abscess in the right frontal lobe and multifocal hypodense lesions in both cerebral hemispheres. Recent post-craniotomy changes were noted in the right frontal region, with small punctate hemorrhagic foci being detected in the surgical bed of the right frontal lobe (Figure 3).

After a month of hospitalization, the patient was discharged home. Oral voriconazole and intravenous amphotericin B were continued for the next two months. The patient could not be followed up in our outpatient department because of affordability issues. Therefore, he continued medications under the care of a public sector hospital. We remained in contact with the patient via telephonic communication. After completing the three-month antifungal course, the patient was re-evaluated. Analysis of the aspirate from the persistent right-sided lesion revealed the recurrence of *Acremonium* species. The patient was started again on intravenous amphotericin B and oral voriconazole for the next three months.

## Discussion

The emergence of many fungi, which were once considered pathogenically insignificant, as new etiological agents of various clinical conditions poses a serious diagnostic and therapeutic challenge. The increased incidence of fungal infections is associated with significant morbidity and mortality [4]. Although *Aspergillus* and *Fusarium* are the two most pathogenic filamentous fungi, the involvement of *Acremonium* species is being increasingly recognized in both localized and systemic infections [3,5]. The genus *Acremonium* is highly polyphyletic and consists of hyaline filamentous molds [1].

In immunocompetent hosts, *Acremonium* species can cause cutaneous and subcutaneous infections, such as superficial skin infections, mycetoma, endophthalmitis, and keratitis, usually after trauma [6]. On the other hand, in immunocompromised individuals, these fungi mainly cause invasive infections, manifesting in the form of osteomyelitis, arthritis, pneumonia, peritonitis, or disseminated infections, which affect a multitude of organ systems [6].

We report the first case of brain abscess caused by *Acremonium* species in a patient with no identifiable co-morbidities in Pakistan. Brain abscess is a serious and potentially life-threatening emergency that requires prompt diagnosis and treatment. Any delay in the management of brain abscesses can lead to poor health outcomes. Both pharmacological and surgical treatments may prove to be very significant in managing fungal brain abscesses [7,8]. However, given the rarity of *Acremonium* infection and the limited published literature available, there are no established guidelines regarding its treatment.

*In vitro* data have revealed that *Acremonium* species are resistant to many antifungal drugs [1,6]. Newer azoles, such as voriconazole and posaconazole, may be effectively used against this fungus [9,10]. However, flucytosine, echinocandins, and fluconazole are not active against it [11,12].

In previous studies, deep tissue infections, such as a right-sided pacemaker-related endocarditis and pneumonia, caused by *Acremonium* species have been successfully treated with voriconazole [8,13]. Mattei et al. reported the successful treatment of two cases of fungemia with voriconazole [12]. Similarly, Herbrecht et al. reported the successful treatment of a case of pulmonary fungal infection with posaconazole [14]. Both voriconazole and posaconazole have high efficacy, good oral bioavailability, and high concentration in tissues, including those of the central nervous system (CNS) [15]. However, the recurrence of fungi has been reported after treatment with voriconazole [16]. In the present case, the use of intravenous amphotericin B and voriconazole initially caused great improvement; however, the recurrence of *Acremonium* species was noted after three months of completion of the antifungal course.

## Conclusions

Although *Acremonium* is normally considered to be present in the environment, it has the potential to cause serious infections in humans. In this case report, we highlight the importance of performing a thorough evaluation for the identification of uncommon infectious diseases. In addition, it is essential for infectious disease specialists, neurosurgeons, and microbiologists to follow a multidisciplinary approach for the effective delivery of care in cases of rare, life-threatening fungal infections involving the CNS. Furthermore, the long-term use of newer azoles along with amphotericin B in cases of invasive fungal infections may result in reduced morbidity.
